# Purified Dendritic Cell-Tumor Fusion Hybrids Supplemented with Non-Adherent Dendritic Cells Fraction Are Superior Activators of Antitumor Immunity

**DOI:** 10.1371/journal.pone.0086772

**Published:** 2014-01-23

**Authors:** Yunfei Zhang, Wen Luo, Yucai Wang, Yunyan Liu, Lianhe Zheng

**Affiliations:** 1 Center of Orthopaedic Surgery, Orthopaedics Oncology Institute of Chinese PLA, Tangdu Hospital, Fourth Military Medical University, Xi’an, Shaanxi Province, China; 2 Department of Ultrasound, Xijing Hospital, Fourth Military Medical University, Xi’an, Shaanxi Province, China; Istituto Superiore di Sanità, Italy

## Abstract

**Background:**

Strong evidence supports the DC-tumor fusion hybrid vaccination strategy, but the best fusion product components to use remains controversial. Fusion products contain DC-tumor fusion hybrids, unfused DCs and unfused tumor cells. Various fractions have been used in previous studies, including purified hybrids, the adherent cell fraction or the whole fusion mixture. The extent to which the hybrids themselves or other components are responsible for antitumor immunity or which components should be used to maximize the antitumor immunity remains unknown.

**Methods:**

Patient-derived breast tumor cells and DCs were electro-fused and purified. The antitumor immune responses induced by the purified hybrids and the other components were compared.

**Results:**

Except for DC-tumor hybrids, the non-adherent cell fraction containing mainly unfused DCs also contributed a lot in antitumor immunity. Purified hybrids supplemented with the non-adherent cell population elicited the most powerful antitumor immune response. After irradiation and electro-fusion, tumor cells underwent necrosis, and the unfused DCs phagocytosed the necrotic tumor cells or tumor debris, which resulted in significant DC maturation. This may be the immunogenicity mechanism of the non-adherent unfused DCs fraction.

**Conclusions:**

The non-adherent cell fraction (containing mainly unfused DCs) from total DC/tumor fusion products had enhanced immunogenicity that resulted from apoptotic/necrotic tumor cell phagocytosis and increased DC maturation. Purified fusion hybrids supplemented with the non-adherent cell population enhanced the antitumor immune responses, avoiding unnecessary use of the tumor cell fraction, which has many drawbacks. Purified hybrids supplemented with the non-adherent cell fraction may represent a better approach to the DC-tumor fusion hybrid vaccination strategy.

## Introduction

Dendritic cell (DC)-tumor fusion hybrids have demonstrated advantages among DC-based tumor vaccination strategies. Using the fusion approach, multiple Tumor associated antigens (TAAs), including those yet unidentified, are endogenously processed by major histocompatibility complex (MHC) I and II pathways in the context of co-stimulatory molecules [1], [2], [3]. Several animal studies and early clinical trials have shown encouraging results from DC and tumor cell fusion [4], [5] [6], [7], [8], [9], [10], [11], [12], [13], [14], [15].

According to previous studies, the fusion efficiency (including electro-fusion and chemical fusion) between DC and tumor cells is relatively low, at less than 50% [2], [16], so the total DC-tumor fusion products contain DC-tumor fusion hybrids, unfused DCs and tumor cells, and DC-DC or tumor-tumor self-fusion, as well as debris and lysate from cells that die during the process. However, the extent to which the hybrids themselves and other components are responsible for inducing anti-tumor immunity is not well understood. In addition, identification of the best components that should be used is controversial, and various fractions from the total fusion products, including purified hybrid cells [8], [9], [16], [17], [18], the adherent cell fraction [2], [19], [20] or the entire fusion mixture [7], [21], [22], [23], have been used in previous studies. To the best of our knowledge, any attempt at fusion requires DCs and tumor cells to be mixed together, so potential co-stimulation and antigen presentation is possible even if no fusion occurs. Thus, it is difficult to know whether reported therapeutic responses result from the presence of a fused DC-tumor component or from unfused DCs presenting antigen through uptake of tumor-associated material or other components in the fusion mixture.

In order to investigate the roles of hybrids themselves and other fusion product components in anti-tumor immunity and to determine which components should be used in the DCs-tumor fusion vaccination, patient-derived DCs and auto breast tumor cells were electro-fused to generate the fusion hybrids and then fluorescence activated cell sorting FACS was used to purify the truely fused cells. We then compared the antitumor immune responses induced by purified hybrids to that of other components in the total fusion mixture. The results showed that except for the DC-tumor hybrids, which play the key role in the antitumor immunity, the non-adherent cell fraction, mostly containing unfused DCs, have a large contribution to antitumor immunity. The cytotoxic T lymphocyte (CTL) assays showed that purified hybrid cells supplemented with the non-adherent cell population can elicit the most effective lysis. Thus, the unfused DCs should also be taken into account during fusion hybrid research.

We further explored the mechanism of immunogenicity from unfused DC in non-adherent cell fraction. For the first time, we showed that unfused DCs can phagocytose apoptotic/necrotic tumor cells or tumor cell debris and then undergo maturation, which may be the main reason why the non-adherent cell population consisting of mainly unfused DCs was able to elicit effective antitumor immunity. We further found it is the DCs with phagocytic tumor cells that played the key role in the antitumor immune responses from the non-adherent unfused DCs. Our study may provide the experimental basis for the use of purified hybrid cells supplemented with the non-adherent cell fraction instead of purified hybrids alone, the adherent cell fraction alone, or the total fusion mixture.

## Materials and Methods

Ethics Statements: The experiment related to human peripheral blood and tumor tissue. We stated that we got the approval of IRB for human participants of Fourth military medical university and Tangdu Hospital. All the participants provided their written informed consent to participate in this study and ethics committees approved this consent procedure.

### 1. Generation of Patient-derived DCs

PBMC-derived DCs from 4 patients with breast cancer were generated as previously described [24]. Granulocyte macrophage colony-stimulating factor (GM-CSF; 100 ng/ml; R&D), interleukin-4 (IL-4; 50 ng/ml; R&D) and tumor necrosis factor α (TNF α; 1000 U/ml; R&D) were used to culture DCs. DCs were harvested from the non-adherent and loosely adherent cells, and the firmly adherent monocytes were harvested after treatment with trypsin and used as an autologous target in the CTL assay.

### 2. Preparation of Autologous Tumor Cells and Tumor Cell Lines

Fresh breast cancer tumor cells were obtained from surgical specimens from 4 patients. Single-cell suspensions were obtained by processing solid tumor samples under sterile conditions as previously described [25]. Briefly, the surgical specimens were mechanically and enzymatically dissociated to generate a single cell suspension, which was used as the fusion partner and as targets for CTL assay. Normal breast tumor lines MCF7, SKBR3 and BT20 and K562 cell line (originated from pleural fluid of leukemia patients) were obtained from American Type Culture Collection (ATCC).

### 3. Fluorescence Dye Stain and Electro-fusion of DCs and Auto Breast Tumor Cells

DCs and auto breast tumor cells were pre-stained red and green, respectively, using PKH26-GL and PKH67-GL Kits (Sigma) according to the manufacturer’s instructions. Electro-fusion of DCs and tumor cells was performed as described previously [2]. Briefly, DCs were mixed with tumor cells (irradiated 5000 cGy) at a ratio of 2∶1 in the fusion medium. After centrifugation, the pellets were resuspended in the same fusion medium without BSA at a concentration of 1×10^7^ cells/ml. Electro-fusion was carried out using a custom designed concentric fusion chamber connected to a pulse generator (ECM 2001,BTX Instrument, Genetronics, San Diego, CA). After fusion, the cells were suspended in the DC medium and incubated overnight at 37°C with 5% CO_2_.

### 4. Enrichment of Hybrid Cells Through FACS Sorting

After overnight culture, the adherent cell population and the non-adherent cell population were harvested separately. The adherent cell population was collected and resuspended in PBS at a concentration of 1×10^7^ cells/ml for sorting. The hybrid cells (dual color) were gated and sorted using a FACSCalibur cell sorter (FACSAria^TM^; BD Biosciences, San, Jose, CA USA). The sorted cells, displaying both green and red fluorescence, were harvested and resuspended in medium for in vitro stimulation. The adherent cell population deprived of hybrid cells, which consisted of mainly tumor cells or tumor-tumor self-fusion (adherent tumor cells), were harvested for in vitro stimulation. The hybrid cell percentage in the adherent cell populationah or purified hybrids population were verified using two-color flow cytometry.

### 5. T Cell Proliferation Assay

Standard [^3^H] thymidine incorporation was used to determine the T cell proliferation that was induced by hybrids themselves or other components of the fusion products. Briefly, non-adherent PBMCs from the same patient were purified through nylon wool to remove antigen-presenting cells and B cells. They were incubated with purified hybrid cells, adherent tumor cells, the non-adherent cell fraction, the adherent cell fraction, purified hybrid cells supplemented with the non-adherent cell fraction, the total fusion product fraction, DCs mixed with tumor cells, or DCs at a ratio of 10∶1 in the presence of 20 units/ml human IL-2 in medium containing 10% human serum. Non-adherent PBMCs cultured in the presence of 20 units/mL human IL-2 was used as control. On day 5, 1 µCi (0.037 MBq) [^3^H] thymidine was added to each well, then cells were harvested 18 hours later and the proliferation was evaluated based on incorporated [^3^H] thymidine tested using liquid scintillation. All determinations were conducted in triplicate and data is expressed as the mean± SD.

### 6. IFN-gamma Enzyme-linked Immunosorbent Spot Assay

To determine the IFN-gamma production of T cells induced by hybrids themselves or other components of the fusion products, a human IFN-gamma ELISPOT kit (R&D) was used according to the manufacturer’s instructions. Briefly, non-adherent PBMCs purified using nylon wool were co-cultured with purified hybrid cells, adherent tumor cells, the non-adherent cell fraction, the adherent cell fraction, purified hybrid cells supplemented with the non-adherent cell fraction, the total fusion product fraction, or DCs were mixed with tumor cells at a ratio of 10∶1 for 7 days and then harvested using nylon wool separation. These purified PBMCs were used as effector cells and auto breast tumor cells were used as stimulator cells. Resulting spots were counted using a stereomicroscope under 20× to 40× magnification. Medium alone and tumor cells without effector cells were included as negative controls.

### 7. CTL Assay

CTL assays against auto breast tumor cells induced by the hybrids themselves or other components of the fusion products were performed using a CytoTox 96 Non-Radioactive Cytotoxicity Assay kit (Promega, ) according to the manufacturer’s instructions. Briefly, non-adherent PBMCs purified using nylon wool were stimulated with purified hybrid cells, adherent tumor cells, the non-adherent cell fraction, the adherent cell fraction, purified hybrid cells supplemented with the non-adherent cell fraction, the whole fusion product fraction, or DCs mixed with tumor cells for 7 days in the presence of 20 units/ml human IL-2.These PBMCs were used as the effector T cells. Then auto breast tumor cells were co-cultured with the effector T cells for 4 h at 1∶12.5, 1∶25, and 1∶50 ratio. The absorbance values were measured at 492 nm. The percentage of cytotoxicity for each effector: target cell ratio was calculated from the equation: [A (experimental) − A (effector spontaneous) − A (target spontaneous)]×100/[A (target maximum) − A (target spontaneous)]. To test whether the cytotoxicity is breast tumor cell-specific, normal breast tumor cells lines MCF7, SKBR3 and BT20, natural killer-sensitive K562 cells and monocytes from the same patient were used as targets in a parallel CTL assay as controls. The ratio for effector cells and K562 cells or monocytes was 50∶1.

### 8. Tumor Cell Apoptosis/Necrosis Analysis after Irradiation and Electro-fusion

Auto breast tumor cells (from patient1 irradiated using 5000 cGy) were suspended in fusion medium to analyze the tumor cell apoptosis/necrosis during electro-fusion. Electro-fusion was then carried out using the same process as for fusion between DC and tumor cells. After fusion, the cells were incubated overnight at 37°C with 5% CO_2_. Apoptosis and necrosis were then assessed using Annexin-V and Propidium iodide (PI) binding (Annexin-V apoptosis detection kit, BD Biosciences, San José, CA) and Flow Cytometric (FACS) analysis. Auto breast tumor cells without irradiation and electro-fusion were used as controls.

### 9. DC Phagocytosis of Apoptotic/Necrotic Tumor Cells during DC/Tumor Fusion Process

In order to analyze whether DCs can phagocyte apoptotic tumor cells during electro-fusion, fluorescence dye staining and electro-fusion of DCs and auto breast tumor cells (from patient1)were performed as described previously. DCs mixed with tumor cells was used as a control. After overnight incubation, the non-adherent cell fraction, which mainly consists of unfused DCs, was harvested and then FACS analysis was performed. DC phagocytosis of apoptotic/necrotic cells was determined by the percentage of double-positive cells.

### 10. Enhanced DC Maturation in the Non-adherent Cell Fraction in DC/Tumor Electro-fusion Products

In order to stimulate naïve T cells, DCs must become mature and increase the expression of HLA Class II molecules and co-stimulatory signals at the cell surface that are necessary to trigger T cell priming. We next assessed the expression of MHC class II and costimulatory molecules on DCs (from patient1) in the non-adherent cell fraction of the DC/tumor fusion products. The DCs mixed with tumor cells were used as a control.

### 11. The Antitumor Immune Responses of DCs with Phagocytic Tumor cells in Non-adherent Cell Fraction

The non-adherent cell fraction contain DCs and DCs with phagocytic tumor cells. In order to test which component in this non-adherent cell fraction was actually functioned in promoting antitumor immune response, we sorted the DCs and DCs with phagocytic tumor cells in the non-adherent cells fraction and compare the antitumor immune response through Ellispot and CTL assay. Purified hybrids cells or Medium were used as control.

### 12. Statistical Analysis

A one-way ANOVA and followed by the least standard difference (LSD) post hoc test were used to determine the difference within each T cell proliferation assays group, IFN-gamma ELISPOT assay and cytotoxicity assay. χ^2^ was used to determine the difference within tumor apoptosis/necrosis and DCs phagocytosis test. SPSS 10.0 was used to analyze statistical significance. A p value <0.05 was considered significant.

## Results

### 1. Electro-fusion of Auto DCs and Breast Tumor Cells Sorted Using FACS

**Figure 1:**
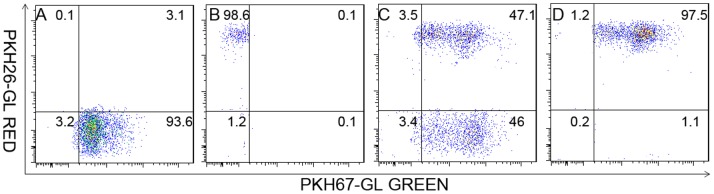
FACS analysis of electro-fusion between auto DCs and tumor cells. Prior to fusion, DCs and tumor cells were stained red and green (from patient 4), respectively, using the PKH26-GL and PKH67-GL kits. Tumor cells were then irradiated with 5000 cGy, and standard electro-fusion was performed on the DCs and tumor cells at a ratio of 2∶1. The fusion mixture was incubated overnight in a CO_2_ tissue culture incubator. On the second day, cells were collected and subjected to FACS analysis. The red DCs and green tumor cells were clearly distinct(A, B). The amount of double-positive hybrid cells in adherent cell population was 47% (C), and 97% in the purified hybrids cell population (D).

After fluorescent-dye staining and electro-fusion and FACS sorting, the percentage of hybrid cells in the adherent cells fraction and purified hybrids cell fraction were verified using two-color flow cytometry. As shown in Fig. 1 (from patient4), the red DCs and green tumor cells were clearly distinct (Fig. 1A, B). After fusion, the percentage of dual-colored cells in the adherent cell population was 47% (Fig. 1C). The dual-colored cells in the adherent cell population were then gated and sorted as purified fusion hybrids. The dual-colored cell percentage in the purified hybrids cell population was 97% (Fig. 1D). The non-adherent cell population and adherent tumor cells were also harvested. T cells from patient4 were HLAA2+/A11− and auto breast tumor cells expressed high level tumor antigen HER2 (Human epidermal growth factor receptor) (data not show).

### 2. T Cell Proliferation Induced by DC-breast Tumor Fusion Cells

**Figure 2:**
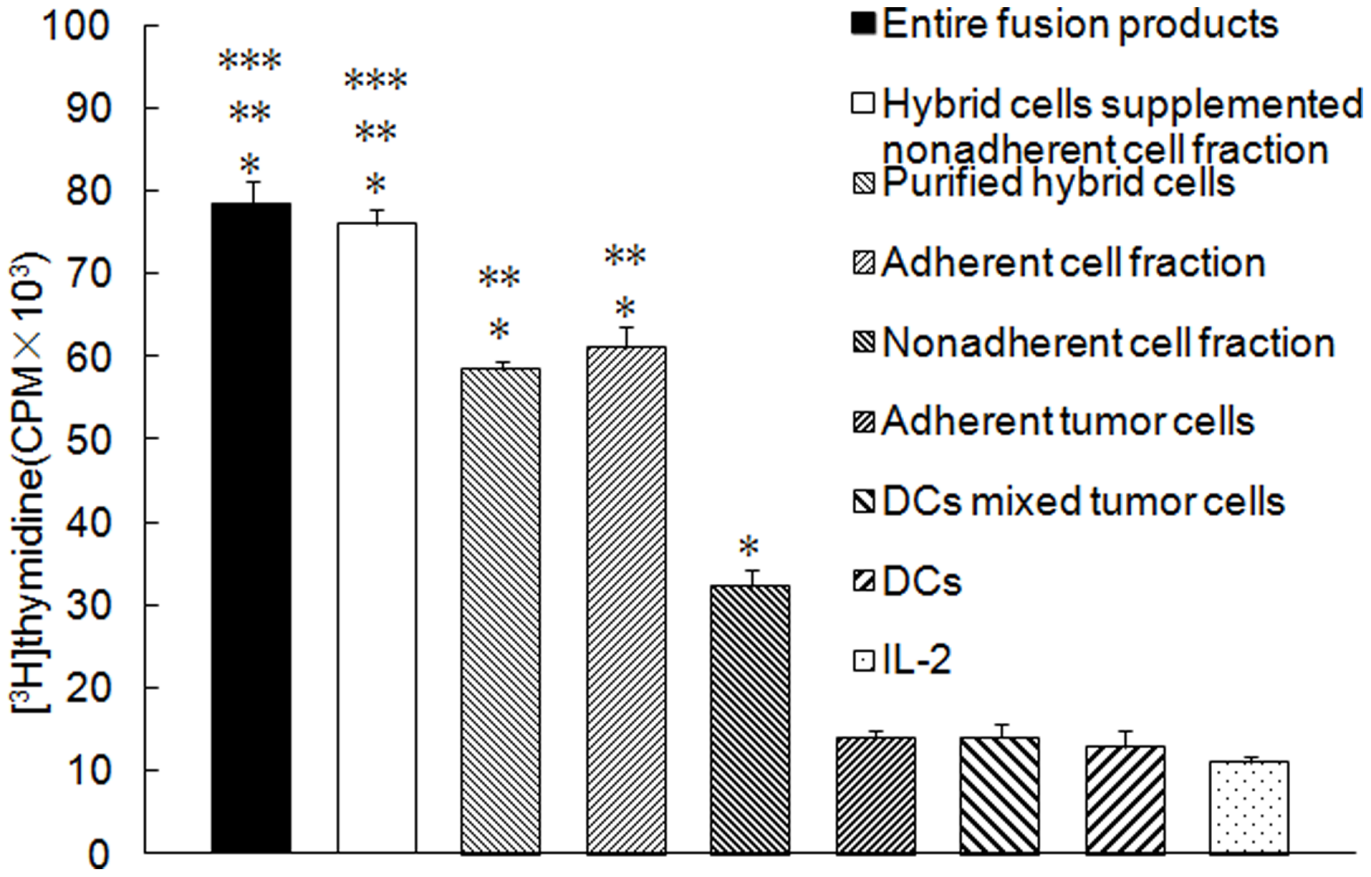
T cells Proliferation by purified DC-tumor hybrids or other components of the fusion products. Non-adherent PBMCs from the same patient (from patient1) were purified using nylon wool to remove antigen-presenting cells and B cells. They were incubated with purified hybrid cells, adherent tumor cells, the non-adherent cell fraction, the adherent cell fraction, purified hybrid cells supplemented with non-adherent cell fraction, total fusion products, DCs mixed with tumor cells, or DCs at a ratio of 10∶1 in the presence of 20 units/ml human IL-2. T cells cultured in the presence of 20 units/ml human IL-2 were used as a control. T cell proliferation was determined using the standard [^3^H] thymidine uptake assay. *Columns*, mean values of triplicate samples; *bars*, SD. *P<0.05 for T cell proliferation stimulated by purified hybrids, the adherent cell fraction, the non-adherent cell fraction, total fusion products or purified hybrid cells supplemented with the non-adherent cell fraction compared with adherent tumor cells, DCs mixed with tumor cells, DCs, or IL-2 alone. **P<0.05 for T cell proliferation stimulated by the total fusion products, purified hybrid cells supplemented with the non-adherent cell fraction, purified hybrid cells or the adherent cell fraction compared with the non-adherent cell fraction. ***P<0.05 for T cell proliferation stimulated by the total fusion products or purified hybrid cells supplemented with the non-adherent cell fraction compared to purified hybrid cells or the adherent cell fraction. There was no difference between the total fusion products and purified hybrid cells supplemented with the non-adherent cell fraction or between purified hybrids and the adherent cell fraction (*P*>0.05).

As shown in Fig. 2 (from patient1), purified hybrids, the adherent cell fraction, the non-adherent cell fraction, total fusion products, and purified hybrid cells supplemented with the non-adherent cell fraction significantly induced T cell proliferation (*P*<0.05) compared to adherent tumor cells, DCs mixed with tumor cells, DCs, or IL-2 alone. In addition, total fusion products or purified hybrid cells supplemented with the non-adherent cell fraction induced the highest T cell proliferation. Purified hybrid cells or the adherent cell fraction elicited more effective T cell proliferation than did the non-adherent cell fraction (*P*<0.05). There was no difference between total fusion products and purified hybrid cells supplemented with the non-adherent cell fraction or between purified hybrids and the adherent cell fraction (*P*>0.05).

### 3. IFN-gamma Production of T cells by DC-tumor Fusion Cells

**Figure 3:**
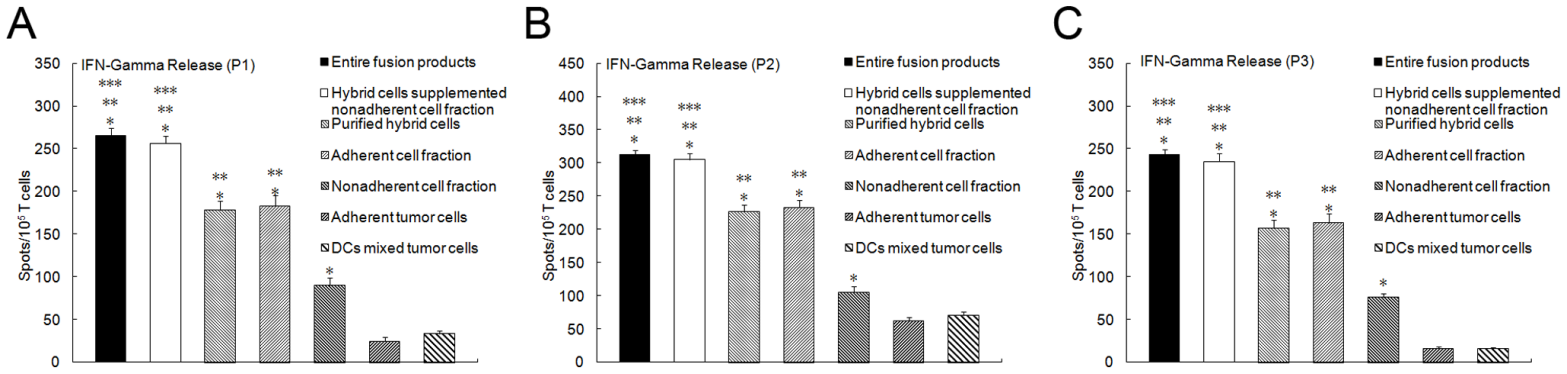
IFN-gamma production stimulated with purified DC/tumor hybrids or other components of the fusion products. Non-adherent PBMCs, purified using nylon wool, were co-cultured with purified hybrid cells, adherent tumor cells, the non-adherent cell fraction, the adherent cell fraction, purified hybrid cells supplemented with the non-adherent cell fraction, total fusion products, or DCs mixed with tumor cells at a ratio of 10∶1 in complete RPMI 1640 for 7 days and then harvested as effector cells using nylon wool separation. Patient-derived tumor cells were used as stimulator cells. IFN-gamma production was determined using a human IFN-gamma ELISPOT kit. A medium-only control and tumor cells without effector cells were included as negative controls. *Columns*, mean values of triplicate samples; *bars*, SD. *P<0.05 for the number of IFN-gamma positive T cells induced by purified hybrids, the adherent cell fraction, the non-adherent cell fraction, total fusion products or purified hybrid cells supplemented with the non-adherent cell fraction compared to DCs mixed with tumor cells. **P<0.05 for the number of IFN-gamma positive T cells induced by total fusion products, purified hybrid cells supplemented with the non-adherent cell fraction, purified hybrid cells or the adherent cell fraction compared with non-adherent cell fraction. ***P<0.05 for the number of IFN-gamma-positive T cells induced by total fusion products or purified hybrid cells supplemented with the non-adherent cell fraction compared with purified hybrid cells or the adherent cell fraction. There was no difference between total fusion products and purified hybrid cells supplemented with the non-adherent cell fraction or between purified hybrids and the adherent cell fraction (*P*>0.05). (A from patient1, B from patient2, C from patient3).

Stimulation by auto breast tumor cells caused higher IFN-gamma secretion by T cells activated by purified hybrids, the adherent cell fraction, the non-adherent cell fraction, total fusion products, and purified hybrid cells supplemented with the non-adherent cell fraction than by adherent tumor cells, or DCs mixed with tumor cells (*P*<0.05; Fig. 3 patient 1, patient2, patient3). Total fusion products or purified hybrid cells supplemented with the non-adherent cell fraction induced the highest IFN-gamma production. Purified hybrid cells or the adherent cell fraction induced more IFN-gamma production than the non-adherent cell fraction (*P*<0.05). There was no difference between total fusion products and purified hybrid cells supplemented with the non-adherent cell fraction or purified hybrids and the adherent cell fraction (*P*>0.05).

### 4. CTL Responses Induced by DC-tumor Fusion Cells

**Figure 4:**
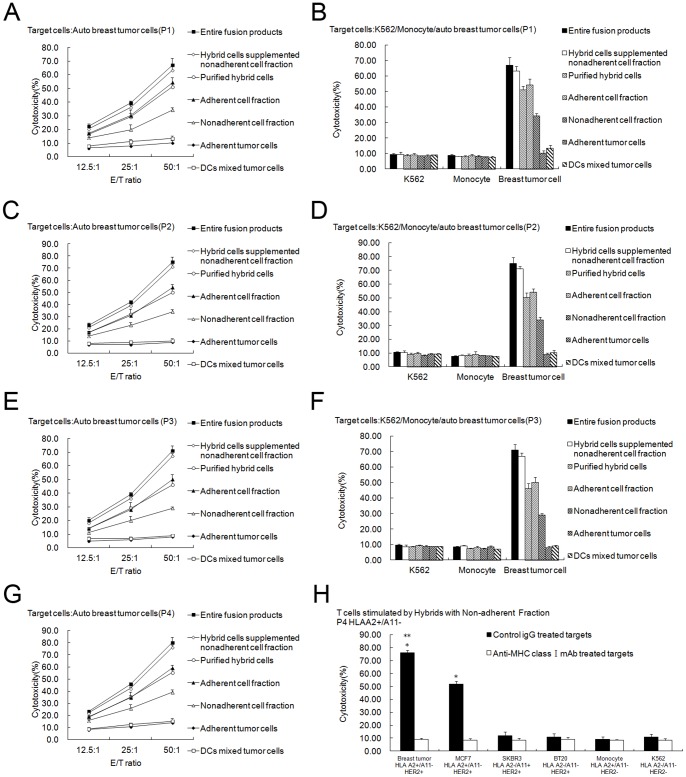
CTL assays induced by purified DC-tumor hybrids or other components of the fusion products. The cytotoxicity assays were performed using CytoTox 96 Non-Radioactive Cytotoxicity Assay kit. (A, C, E, G) Non-adherent PBMCs stimulated with total fusion products (▪), hybrid cells supplemented with the non-adherent cell fraction (◊), purified hybrid cells (○), the adherent cell fraction (▴), the non-adherent cell fraction (△), adherent tumor cells (♦), or DCs mixed with tumor cells (□) for 7 days in the presence of 20 units/mL human IL-2 were used as the effector T cells. Then breast tumor cells were co-cultured with the effector T cells for 4 h at ratios of 1∶12.5, 1∶25, and 1∶50, respectively. *Points*, mean values of triplicate samples; *bars*, SD. The results showed that T lymphocytes activated by purified hybrids, the adherent cell fraction, the non-adherent cell fraction, total fusion products, purified hybrid cells supplemented with the non-adherent cell fraction lysed auto breast tumor cells much more effectively than T cells activated by adherent tumor cells or DC mixed with tumor cells (*P*<0.05). Lysis induced by total fusion products or purified hybrid cells supplemented with the non-adherent cell fraction was the most effective. Purified hybrid cells or the adherent cell fraction induced more effective lysis than the non-adherent cell fraction (*P*<0.05). There was no difference between total fusion products and purified hybrid cells supplemented with the non-adherent cell fraction or between purified hybrids and the adherent cell fraction (*P*>0.05). (B, D, F) Natural killer-sensitive K562 cells and monocytes were used as control targets in a parallel CTL assay, and the ratio for effector and target cells was 50∶1. *Columns*, mean values of triplicate samples; *bars*, SD. No lysis against K562 or monocytes was induced. H, T cells were stimulated by purified hybrid cells supplemented with the non-adherent cell fraction and then normal breast tumor cells lines MCF7, SKBR3 and BT20 were included as targets and MHC Class I molecule blocking test was done. Effector T can not only lyse the auto breast tumor cells (HLA A2+/A11−, HER2+), but also lyse the HLA-A2 matched MCF7 (HLA A2+/A11−, HER2+) to a less extent ( P<0.05) which can be blocked by preincubation with anti-MHC I antibody. (A B from patient1, C D from patient2, E F from patient3, G H from patient4).

Auto breast tumor cells were more effectively lysed by T lymphocytes activated by purified hybrids, the adherent cell fraction, the non-adherent cell fraction, total fusion products, and purified hybrid cells supplemented with the non-adherent cell fraction compared to T cells activated by adherent tumor cells or DCs mixed with tumor cells (*P*<0.05; Fig. 4A from patient1, C from patient2, E from patient3, G from patient4 (HLAA2+/A11−). In addition, the lysis induced by total fusion products or purified hybrid cells supplemented with the non-adherent cell fraction was the most effective. Purified hybrid cells or the adherent cell fraction induced more effective lysis than the non-adherent cell fraction (*P*<0.05). There were no differences between total fusion products and purified hybrid cells supplemented with the non-adherent cell fraction or between purified hybrids and the adherent cell fraction (*P*>0.05).

The lysis was auto breast tumor cell-specific because no lysis of natural killer-sensitive K562 cells or monocytes was observed (Fig. 4B from patient1, D from patient2, F from patient3). Furthermore, we found less level of lysis against other tumor targets was also observed. As figure 4H showed, effector T cells (HLA A2+/A11− from patient4) stimulated by purified hybrid cells supplemented with the non-adherent cell fraction can not only lyse the auto breast tumor cells (HLA A2+/A11−, HER2+), but also lyse the HLA-A2 matched MCF7 (HLA A2+/A11−, HER2+) to a less extent ( P<0.05). However, the lysis was much less against SKBR3 (HLA A2−, HER2+) or BT20 (HLA A2−, HER2+). In addition, lysis of the targets was abrogated by preincubation of the tumor cells with anti-HLA-ABC mAb (Figure 4H). These results indicated that the cytotoxicity of CTLs induced by purified hybrid cells supplemented with the non-adherent cell fraction may be tumor antigen specific and restricted by MHC Class I molecule. However, the present data is insufficient to completely address the tumor specificity and HLA restriction of the CTL and more studies will be performed in the future.

### 5. Gamma-irradiation and Electro-fusion Induced Tumor Cell Apoptosis

**Figure 5:**
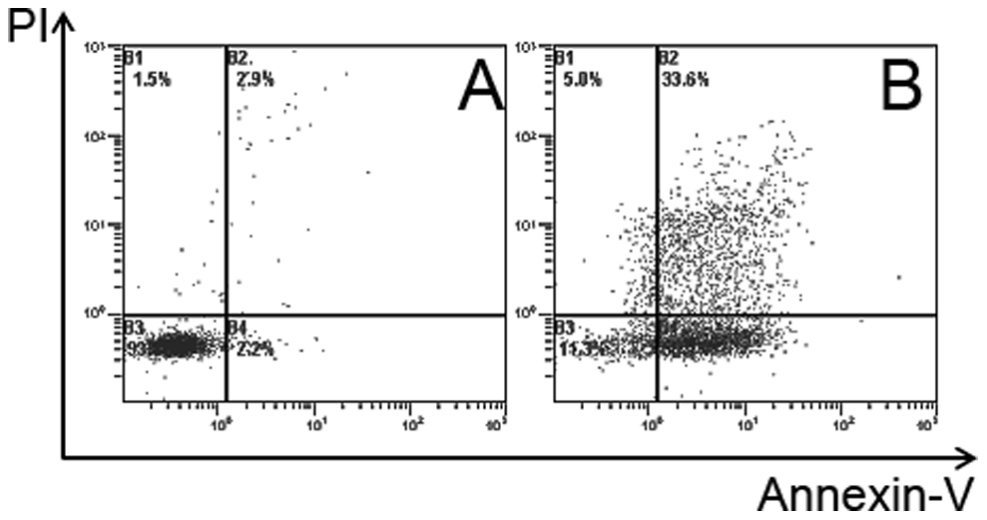
Apoptosis/necrosis of tumor cells induced by gamma-irradiation and electro-fusion. After undergoing irradiation and electro-fusion, tumor cell (from patient1) apoptosis/necrosis was assessed using the Annexin-V /PI analysis. *A,* Apoptosis/necrosis analysis of tumor cells without irradiation and electro-fusion. No significant apoptosis or necrosis was observed. *B*, Tumor cells (33.6%) developed apoptosis/necrosis after gamma-irradiation and electro-fusion. Early apoptotic cells were defined as annexin V-FITC+/PI−, while necrotic cells were double-positive.

After irradiation with 5000 cGy, auto breast tumor cells (from patient1) underwent the electro-fusion process. After fusion and overnight incubation, apoptosis and necrosis were assessed using Annexin-V/PI analysis. One-third (33.6%) of tumor cells developed necrosis characterized by Annexin-V+/PI+ staining (Fig. 5A). In contrast, no significant apoptosis or necrosis was observed in tumors without irradiation and electro-fusion, as shown in Fig. 5B.

### 6. DCs Efficiently Phagocytose Apoptotic/Necrotic Tumor Cells

**Figure 6:**
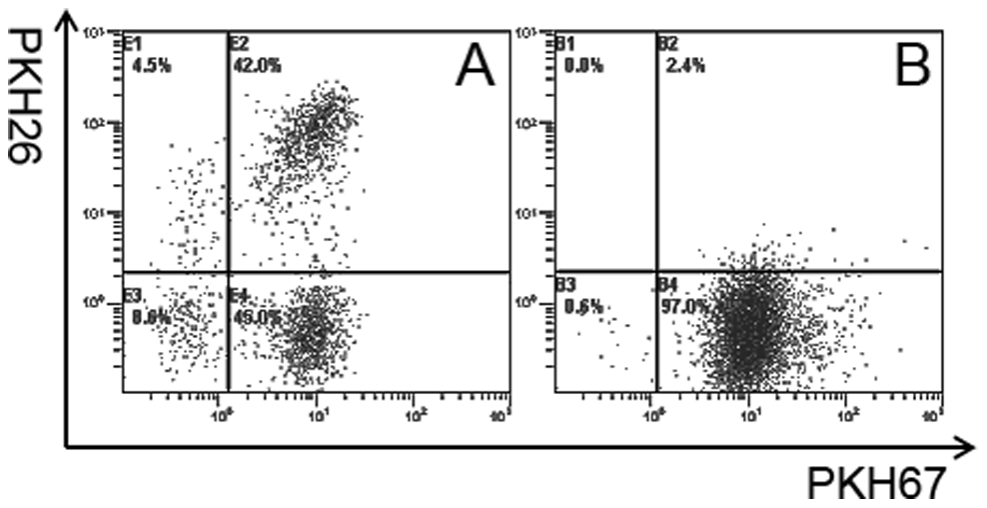
FACS analysis of apoptotic/necrosis tumor cells phagocytosed by DCs. DCs and auto breast tumor cells (from patient 1) were stained red and green by PKH26 and PKH67 and double positive cells were analyzed using FACS and confocal microscopy. A, FACS analysis of the non-adherent cells fraction from the total fusion products. DC phagocytosis of apoptotic tumor cells was calculated as the percentage of double-positive cells, and was approximately 42%. B, FACS analysis of DCs mixed with tumor cells. No significant phagocytosis was observed.

PKH26 red-labeled DCs were electro-fused with PKH67 green-labeled tumor cells (from patient1), as described previously. After an overnight incubation, the non-adherent fraction containing mainly unfused DCs was harvested. The amount of DCs that had phagocytosed apoptotic/necrotic tumor cells was calculated as the percentage of double-positive cells; this percentage was 42% (Fig. 6A). No significant phagocytosis was observed when DCs were mixed with tumor cells (Fig. 6B).

### 7. Phagocytosis of Apoptotic/Necrotic Tumor Cells Induces DC Maturation

**Figure 7:**
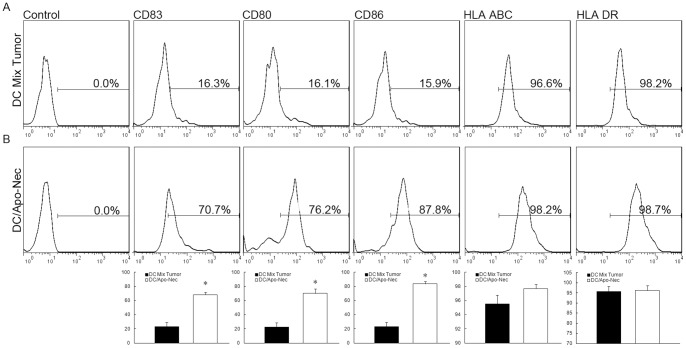
DC maturation analysis after phagocytosis of apoptotic/necrotic tumor cells. The non-adherent cell fraction from DC/tumor fusion products or from the DC tumor mixture (from patient1) was stained with fluorescence labeled antibody against MHC class II, CD80, CD86 and CD83 and FACS analysis was performed. A, Non-adherent cells (containing mainly unfused DCs) from the total fusion products demonstrated significant MHC class II (increased fluorescence intensity), CD80, CD86 and CD83 up-regulation. B, Up-regulation of these molecules in DCs cocultured with tumor cells was minimal. In each histogram the percentage of positive cells is indicated.

In order to stimulate naïve T cells, DCs must become mature, increasing the expression of HLA Class II molecules and co-stimulatory signals at the cell surface that are necessary to trigger T cell priming. As observed in Figure 7 (from patient 1), phagocytosis of apoptotic/necrotic cells induced DCs maturation compared to DCs mixed with tumor cells. DCs from the non-adherent cell fraction in the DC/tumor electro-fusion products had significant up-regulation of MHC class II as well as CD80, CD86 and CD83. By contrast, up-regulation of these molecules in DCs cocultured with tumor cells was minimal. There was a statistical significance in DC maturation between the two groups.

### 8. Effective Antitumor Immune Responses of DCs with Phagocytic Tumor Cells in Non-adherent Cell Fraction

**Figure 8:**
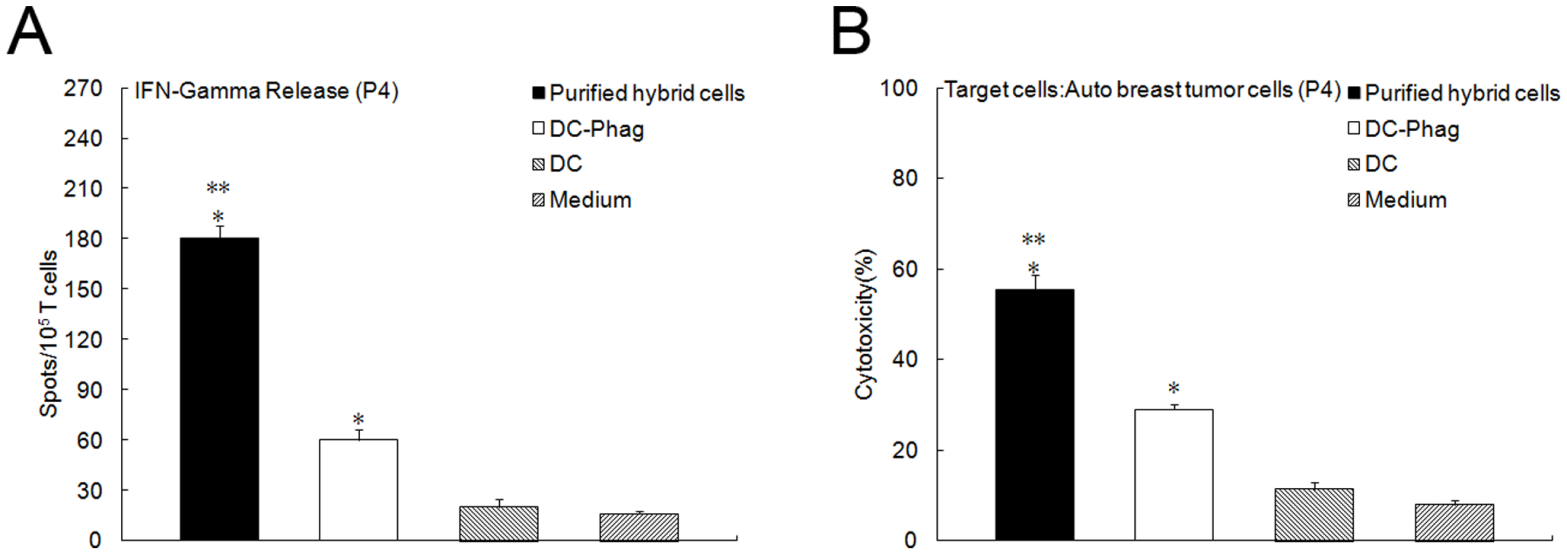
Antitumor immune responses of DCs with phagocytic tumor cells in non-adherent cell fraction. DCs and DCs with phagocytic tumor cells in the non-adherent cells fraction were sorted and antitumor immune response were compared through Ellispot and CTL assay (from patient4). Compared with DCs, DCs with phagocytic tumor cells can stimulate more IFN-Gamma secretion (A, P<0.05) and much more powerful CTL responses (B, P<0.05) and the immune responses were not as strong as fusion hybrids (P<0.05).

The non-adherent cells fraction contained DCs and DCs with phagocytic tumor cells and antitumor immune responses were compared through Ellispot and CTL assay. As showed in Figure 8 (from patient4), compared with DCs, DCs with phagocytic tumor cells can stimulate more IFN-Gamma secretion and much more powerful CTL responses (Although the immune responses were not as strong as fusion hybrids). It is indicated that in the non-adherent cells fraction, it is the DCs with phagocytic tumor cells that play the key role in the antitumor immune responses.

## Discussion

Despite the strong preclinical evidence supporting the use of DC/tumor fusions for cancer vaccination, conflicting results have been observed to date in clinical trials [26], [27], [28], [29], [30]. One possible reason for this controversy is that the data supporting the formation of fusion cells that were used in the clinical trials is not definitive [31] and the level of fusion efficiency is relatively low and variable. Another possible explanation is that immunosuppressive substances such as TGF-β, which is derived from tumor cells, was used to prepare the fusion cells [32]. Tumor-derived TGF-β participates in tumor immune escape by suppressing the host’s CTL function, which is a critical requirement for killing tumor cells [33]. Kao et al showed that tumor-derived TGF-β significantly reduces the ability of DC/tumor fusion cells to prime antitumor immunity and TGF-β-blocking strategies can enhance antitumor immunity [32], [34]. Thus, the tumor cells and tumor-tumor fusion cell fraction of the total fusion products is not suggested for use in the DC/tumor fusion strategy. However, the components that should be used are controversial, and various fractions from the total fusion products have been chosen and used in previous studies, including purified hybrid cells [8], [9], [16], [17], [18], the adherent cell fraction [2], [19], [20] or the entire fusion mixture [7], [21], [22], [23].

The total DC-tumor fusion product mixture contain DC-tumor fusion hybrids, unfused DCs and tumor cells and DC-DC or tumor-tumor self fusion as well as debris and lysate from cells that die during the process. Thus, it remains to be determined the extent to which the hybrids themselves are responsible for the induction of antitumor immunity, the extent to which other components of the product may contribute, and which components should be used to maximize the antitumor immunity.

In our study, fusion cells were generated between DCs from patients and patient-derived auto breast tumor cells. We purified the DC-tumor hybrids from the adherent cell fraction using FACS sorting, and harvested the non-adherent cell fraction and adherent tumor cells. Then the antitumor immunity induced by purified hybrids, the adherent cell fraction, the non-adherent cell fraction, total fusion products, purified hybrid cells supplemented with the non-adherent cell fraction and DCs mixed with tumor cells were examined. The results showed that, compared to DCs mixed with tumor cells, DC-tumor hybrid cells or the non-adherent cell fraction was able to induce more efficient antitumor immune responses that were auto breast tumor cell-specific, while the adherent tumor cells did not induce any efficient responses. In addition, purified DC-tumor hybrid cells supplemented with the non-adherent cell fraction and total fusion products were more efficient than the adherent cell fraction or purified hybrid cells alone. Furthermore, the cytotoxicity induced by purified hybrid cells supplemented with the non-adherent cell fraction may be tumor antigen specific and restricted by MHC Class I molecule. Thus, our results showed that, in the total electro-fusion products, except for DC-tumor hybrid cells that play a key role in antitumor immunity, the non-adherent cell fraction, which contains mainly DCs, was a main contributor to antitumor immunity. In addition, purified DC-tumor hybrid cells supplemented with the non-adherent cell fraction were able to maximize antitumor immunity. Holmes *et al*. [17] used human tumor cells fused to autologous DCs with PEG to conduct a side-by-side comparison of the ability of the un-fractionated fusion product (10% hybrids) and FACS-purified hybrids (>95% purity) to induce tumor-specific CTLs in vitro. The purified hybrids stimulated the highest level of CTL activity (∼70% specific lysis), but substantial cytolytic activity was also elicited by the un-fractionated fusion product (∼50% specific lysis), which contained a disproportionately lower percentage of hybrids. These results indicated that the hybrids were particularly potent but other components of the preparation may add to their activity; these results support our findings that, except for DC-tumor electro-fusion hybrid cells, the non-adherent cell fraction containing mainly DCs had a large contribution to the antitumor activity.

We investigated why the non-adherent cell fraction containing mainly unfused DCs showed effective antitumor immunity. We showed for the first time that after the irradiation and electro-fusion process, tumor cells underwent apoptosis/necrosis and then unfused DCs phagocytosed the apoptotic/necrotic tumor cells or tumor debris, which resulted in significant DC maturation. This may be the mechanism of the non-adherent cell fraction immunogenicity. Barrio and other researchers showed that human monocyte-derived immature DCs can efficiently cross-present tumor-associated antigens when co-cultured with a mixture of tumor cells that were rendered apoptotic/necrotic by γ irradiation [35], [36], [37], [38], which is consistent with our findings.

It has been shown that an increased dose of irradiation would induce a higher level of apoptosis [19], and the electro-fusion process can also increase apoptosis/necrosis. A high degree of cell death was observed in tumor cell populations over time after exposure to 5000 cGy in our study (data not shown). We found that after irradiation of 5000 cGy and the electro-fusion process, approximately 33% of tumor cells developed necrosis. Several publications have shown that DCs are able to process apoptotic tumor cells or phagocytose tumor cell debris and present tumor-associated antigens [39]. In our study we showed that 42% of unfused DCs phagocytose the apoptotic/necrotic tumor cells. Gottfried and Krause et al also showed that polyethylene glycol-induced fusion, as well as electro-fusion, also gave rise to DCs that phagocytosed apoptotic/necrotic tumor cells [40]. There is now considerable experimental evidence that phagocytosis of apoptotic/necrotic tumor cells causes DC maturation [35], [37]. In addition, we showed that the unfused DCs developed significant maturation compared to the DC/tumor mixture.

The non-adherent cells fraction contains DCs and DCs with phagocytic tumor cells and we found it is the DCs with phagocytic tumor cells that play the key role in the antitumor immune responses. Thus, these unfused DCs that captured apoptotic tumor cells or phagocytosed tumor cell debris may be one of the main reasons why the non-adherent cell population, consisting mainly unfused DCs, was able to elicit efficient antitumor immunity in our study.

In conclusion, we purified the DC-tumor fusion hybrid cells and then analyzed the role that the hybrids themselves played in the induction of antitumor immunity and the contribution that other electro-fusion product components have made. We showed that, except for DC-tumor fusion hybrid cells that play the key role in the antitumor responses, the non-adherent cell fraction containing mainly unfused DCs had a main contribution to anti-tumor activity. Purified fusion hybrids supplemented with the non-adherent cell population elicited the most effective antitumor responses, which may represent a better approach in the DC-tumor fusion hybrid vaccination strategy.

The advantages for the use of purified fusion hybrids supplemented with the non-adherent cell population are as follows: (1) compared to the total fusion product mixture, patients need not receive many unnecessary cells especially the unfused tumor cells, which may suppress the anti-tumor immune responses due to the TGF-β that is derived from tumor cells; and (2) compared to the use of purified hybrids or the adherent cell fraction containing mainly fusion hybrids and tumor cells, purified fusion hybrids supplemented with the non-adherent cell population can induce much stronger anti-tumor immune responses.

In addition, we explored the immunogenicity mechanisms of the non-adherent cell fraction containing mainly DCs. After irradiation and the electro-fusion process, numerous tumor cells developed apoptosis. We also found that unfused DCs can phagocytose apoptotic tumor cells which showed upregulated maturation and effective antitumor responses.

However, it should be noted that this study has examined only the electro-fusion products and not chemical fusion products. Despite this limitation, this study may represent a better approach in the DC-tumor fusion vaccine strategy, especially for clinical studies. Our study suggested that the unfused DC fraction should also be taken into account in fusion hybrid research.

## References

[1] Gong J, Nikrui N, Chen D, Koido S, Wu Z, et al (2000) Fusions of human ovarian carcinoma cells with autologous or allogeneic dendritic cells induce antitumor immunity. J Immunol 165: 1705–1711. 10903782 10.4049/jimmunol.165.3.1705

[2] Parkhurst MR, DePan C, Riley JP, Rosenberg SA, Shu S (2003) Hybrids of dendritic cells and tumor cells generated by electrofusion simultaneously present immunodominant epitopes from multiple human tumor-associated antigens in the context of MHC class I and class II molecules. J Immunol 170: 5317–5325. 12734382 10.4049/jimmunol.170.10.5317 PMC2553207

[3] Koido S, Ohana M, Liu C, Nikrui N, Durfee J, et al (2004) Dendritic cells fused with human cancer cells: morphology, antigen expression, and T cell stimulation. Clin Immunol 113: 261–269. 15507391 10.1016/j.clim.2004.08.004

[4] Koido S, Tanaka Y, Chen D, Kufe D, Gong J (2002) The kinetics of in vivo priming of CD4 and CD8 T cells by dendritic/tumor fusion cells in MUC1-transgenic mice. J Immunol 168: 2111–2117. 11859096 10.4049/jimmunol.168.5.2111

[5] Gong J, Avigan D, Chen D, Wu Z, Koido S, et al (2000) Activation of antitumor cytotoxic T lymphocytes by fusions of human dendritic cells and breast carcinoma cells. Proc Natl Acad Sci U S A 97: 2715–2718. 10688917 10.1073/pnas.050587197 PMC15995

[6] Lindner M, Schirrmacher V (2002) Tumour cell-dendritic cell fusion for cancer immunotherapy: comparison of therapeutic efficiency of polyethylen-glycol versus electro-fusion protocols. Eur J Clin Invest 32: 207–217. 11895473 10.1046/j.1365-2362.2002.00968.x

[7] Wang J, Saffold S, Cao X, Krauss J, Chen W (1998) Eliciting T cell immunity against poorly immunogenic tumors by immunization with dendritic cell-tumor fusion vaccines. J Immunol 161: 5516–5524. 9820528

[8] Hayashi T, Tanaka H, Tanaka J, Wang R, Averbook BJ, et al (2002) Immunogenicity and therapeutic efficacy of dendritic-tumor hybrid cells generated by electrofusion. Clin Immunol 104: 14–20. 12139943 10.1006/clim.2002.5224

[9] Li J, Holmes LM, Franek KJ, Burgin KE, Wagner TE, et al (2001) Purified hybrid cells from dendritic cell and tumor cell fusions are superior activators of antitumor immunity. Cancer Immunol Immunother 50: 456–462. 11761439 10.1007/s002620100218 PMC11034210

[10] Guo G, Chen S, Zhang J, Luo L, Yu J, et al (2005) Antitumor activity of a fusion of esophageal carcinoma cells with dendritic cells derived from cord blood. Vaccine 23: 5225–5230. 16171908 10.1016/j.vaccine.2005.07.080

[11] Galluzzi L, Senovilla L, Vacchelli E, Eggermont A, Fridman WH, et al (2012) Trial watch: Dendritic cell-based interventions for cancer therapy. Oncoimmunology 1: 1111–1134. 23170259 10.4161/onci.21494 PMC3494625

[12] Rosenblatt J, Vasir B, Uhl L, Blotta S, Macnamara C, et al (2011) Vaccination with dendritic cell/tumor fusion cells results in cellular and humoral antitumor immune responses in patients with multiple myeloma. Blood 117: 393–402. 21030562 10.1182/blood-2010-04-277137 PMC3031474

[13] Siders WM, Garron C, Shields J, Kaplan JM (2009) Induction of antitumor immunity by semi-allogeneic and fully allogeneic electrofusion products of tumor cells and dendritic cells. Clin Transl Sci 2: 75–79. 20443871 10.1111/j.1752-8062.2008.00052.x PMC5350795

[14] Avigan DE, Vasir B, George DJ, Oh WK, Atkins MB, et al (2007) Phase I/II study of vaccination with electrofused allogeneic dendritic cells/autologous tumor-derived cells in patients with stage IV renal cell carcinoma. J Immunother 30: 749–761. 17893567 10.1097/CJI.0b013e3180de4ce8

[15] Cho EI, Tan C, Koski GK, Cohen PA, Shu S, et al (2010) Toll-like receptor agonists as third signals for dendritic cell-tumor fusion vaccines. Head Neck 32: 700–707. 19908319 10.1002/hed.21241

[16] Wei YC, Sticca RP, Li J, Holmes LM, Burgin KE, et al (2007) Combined treatment of dendritoma vaccine and low-dose interleukin-2 in stage IV renal cell carcinoma patients induced clinical response: A pilot study. Oncol Rep 18: 665–671. 17671717

[17] Holmes LM, Li J, Sticca RP, Wagner TE, Wei Y (2001) A rapid, novel strategy to induce tumor cell-specific cytotoxic T lymphocyte responses using instant dentritomas. J Immunother 24: 122–129. 11265769

[18] Wei Y, Sticca RP, Holmes LM, Burgin KE, Li J, et al (2006) Dendritoma vaccination combined with low dose interleukin-2 in metastatic melanoma patients induced immunological and clinical responses. Int J Oncol 28: 585–593. 16465362

[19] Trevor KT, Cover C, Ruiz YW, Akporiaye ET, Hersh EM, et al (2004) Generation of dendritic cell-tumor cell hybrids by electrofusion for clinical vaccine application. Cancer Immunol Immunother 53: 705–714. 15048588 10.1007/s00262-004-0512-1 PMC11032919

[20] Lee WT, Shimizu K, Kuriyama H, Tanaka H, Kjaergaard J, et al (2005) Tumor-dendritic cell fusion as a basis for cancer immunotherapy. Otolaryngol Head Neck Surg 132: 755–764. 15886631 10.1016/j.otohns.2005.01.018

[21] Kugler A, Stuhler G, Walden P, Zoller G, Zobywalski A, et al (2000) Regression of human metastatic renal cell carcinoma after vaccination with tumor cell-dendritic cell hybrids. Nat Med 6: 332–336. 10700237 10.1038/73193

[22] Siders WM, Vergilis KL, Johnson C, Shields J, Kaplan JM (2003) Induction of specific antitumor immunity in the mouse with the electrofusion product of tumor cells and dendritic cells. Mol Ther 7: 498–505. 12727113 10.1016/s1525-0016(03)00044-3

[23] Vasir B, Wu Z, Crawford K, Rosenblatt J, Zarwan C, et al (2008) Fusions of dendritic cells with breast carcinoma stimulate the expansion of regulatory T cells while concomitant exposure to IL-12, CpG oligodeoxynucleotides, and anti-CD3/CD28 promotes the expansion of activated tumor reactive cells. J Immunol 181: 808–821. 18566447 10.4049/jimmunol.181.1.808 PMC2938172

[24] Sallusto F, Lanzavecchia A (1994) Efficient presentation of soluble antigen by cultured human dendritic cells is maintained by granulocyte/macrophage colony-stimulating factor plus interleukin 4 and downregulated by tumor necrosis factor alpha. J Exp Med 179: 1109–1118. 8145033 10.1084/jem.179.4.1109 PMC2191432

[25] Zhang Y, Ma B, Zhou Y, Zhang M, Qiu X, et al (2007) Dendritic cells fused with allogeneic breast cancer cell line induce tumor antigen-specific CTL responses against autologous breast cancer cells. Breast Cancer Res Treat 105: 277–286. 17187233 10.1007/s10549-006-9457-8

[26] Koido S, Hara E, Homma S, Fujise K, Gong J, et al (2007) Dendritic/tumor fusion cell-based vaccination against cancer. Arch Immunol Ther Exp (Warsz) 55: 281–287. 18219758 10.1007/s00005-007-0034-6

[27] Gong J, Koido S, Calderwood SK (2008) Cell fusion: from hybridoma to dendritic cell-based vaccine. Expert Rev Vaccines 7: 1055–1068. 18767954 10.1586/14760584.7.7.1055

[28] Koido S, Hara E, Homma S, Namiki Y, Ohkusa T, et al (2009) Cancer vaccine by fusions of dendritic and cancer cells. Clin Dev Immunol 2009: 657369. 20182533 10.1155/2009/657369 PMC2825547

[29] Koido S, Homma S, Hara E, Namiki Y, Ohkusa T, et al (2010) Antigen-specific polyclonal cytotoxic T lymphocytes induced by fusions of dendritic cells and tumor cells. J Biomed Biotechnol 2010: 752381. 20379390 10.1155/2010/752381 PMC2850552

[30] Koido S, Homma S, Takahara A, Namiki Y, Komita H, et al (2011) Immunologic monitoring of cellular responses by dendritic/tumor cell fusion vaccines. J Biomed Biotechnol 2011: 910836. 21541197 10.1155/2011/910836 PMC3085507

[31] Shu S, Zheng R, Lee WT, Cohen PA (2007) Immunogenicity of dendritic-tumor fusion hybrids and their utility in cancer immunotherapy. Crit Rev Immunol 27: 463–483. 18197808 10.1615/critrevimmunol.v27.i5.50

[32] Kao JY, Gong Y, Chen CM, Zheng QD, Chen JJ (2003) Tumor-derived TGF-beta reduces the efficacy of dendritic cell/tumor fusion vaccine. J Immunol 170: 3806–3811. 12646647 10.4049/jimmunol.170.7.3806

[33] Rich S, Seelig M, Lee HM, Lin J (1995) Transforming growth factor beta 1 costimulated growth and regulatory function of staphylococcal enterotoxin B-responsive CD8+ T cells. J Immunol 155: 609–618. 7608539

[34] Zhang M, Berndt BE, Chen JJ, Kao JY (2008) Expression of a soluble TGF-beta receptor by tumor cells enhances dendritic cell/tumor fusion vaccine efficacy. J Immunol 181: 3690–3697. 18714045 10.4049/jimmunol.181.5.3690

[35] Alfaro C, Suarez N, Onate C, Perez-Gracia JL, Martinez-Forero I, et al (2011) Dendritic cells take up and present antigens from viable and apoptotic polymorphonuclear leukocytes. PLoS One 6: e29300. 22206007 10.1371/journal.pone.0029300 PMC3243708

[36] Barrio MM, Abes R, Colombo M, Pizzurro G, Boix C, et al (2012) Human macrophages and dendritic cells can equally present MART-1 antigen to CD8(+) T cells after phagocytosis of gamma-irradiated melanoma cells. PLoS One 7: e40311. 22768350 10.1371/journal.pone.0040311 PMC3388056

[37] von Euw EM, Barrio MM, Furman D, Bianchini M, Levy EM, et al (2007) Monocyte-derived dendritic cells loaded with a mixture of apoptotic/necrotic melanoma cells efficiently cross-present gp100 and MART-1 antigens to specific CD8(+) T lymphocytes. J Transl Med 5: 19. 17448240 10.1186/1479-5876-5-19 PMC1863425

[38] von Euw EM, Barrio MM, Furman D, Levy EM, Bianchini M, et al (2008) A phase I clinical study of vaccination of melanoma patients with dendritic cells loaded with allogeneic apoptotic/necrotic melanoma cells. Analysis of toxicity and immune response to the vaccine and of IL-10 -1082 promoter genotype as predictor of disease progression. J Transl Med 6: 6. 18221542 10.1186/1479-5876-6-6 PMC2265680

[39] Nouri-Shirazi M, Banchereau J, Bell D, Burkeholder S, Kraus ET, et al (2000) Dendritic cells capture killed tumor cells and present their antigens to elicit tumor-specific immune responses. J Immunol 165: 3797–3803. 11034385 10.4049/jimmunol.165.7.3797

[40] Gottfried E, Krieg R, Eichelberg C, Andreesen R, Mackensen A, et al (2002) Characterization of cells prepared by dendritic cell-tumor cell fusion. Cancer Immun 2: 15. 12747760

